# Trends in the Epidemiology and Treatment of Pediatric-Onset Multiple Sclerosis in Alberta, Canada

**DOI:** 10.1177/08830738231176588

**Published:** 2023-05-18

**Authors:** Camille Yearwood, Colin Wilbur

**Affiliations:** 1 3158 University of Alberta, Edmonton, Canada; 2 504420 Women and Children's Health Research Institute, Edmonton, Canada

**Keywords:** child health, epidemiology, multiple sclerosis, treatment

## Abstract

**Background:**

Fingolimod became the first disease-modifying therapy approved by Health Canada for pediatric multiple sclerosis in 2018, but the impact of that approval on treatment patterns in Canada is unknown. The aim of this study was to describe trends in the epidemiology and treatment of pediatric-onset multiple sclerosis in Alberta, Canada.

**Methods:**

This study entailed a retrospective review of administrative health databases using 2 case definitions of multiple sclerosis. Those <19 years of age at a date of diagnosis between January 1, 2011, and December 31, 2020, were included. Incidence and prevalence estimates were calculated and stratified by sex and age cohort. Pharmacy dispenses of disease-modifying therapies were identified.

**Results:**

106 children met one or both case definitions. In 2020, the age-standardized incidence using the 2 case definitions was 0.47 and 0.57 per 100 000, and the age-standardized prevalence was 2.84 and 3.41 per 100 000, respectively. Seventy-nine incident cases were identified, 38 (48%) of whom were dispensed a disease-modifying therapy prior to age 19 years. Injectables accounted for all initial pediatric disease-modifying therapy dispenses prior to 2019, whereas in 2019-2020 injectables accounted for only 3 of 15 (20%) initial dispenses, and instead B-cell therapies were the most common initial disease-modifying therapy (6 of 15, 40%). In 2020, B-cell therapies were the most common disease-modifying therapy dispensed overall (9 of 22 dispenses, 41%) followed by fingolimod (6 of 22, 27%).

**Conclusion:**

The treatment of children with multiple sclerosis in Alberta has evolved, with a rapid shift in 2019 away from injectables to newer agents, although B-cell therapies—not fingolimod—are now most commonly dispensed.

Multiple sclerosis is an autoimmune disease affecting the central nervous system that is an important cause of disability in young adults. Although the peak age of multiple sclerosis onset occurs between the age of 25 and 45 years, approximately 2% to 5% of cases have onset in childhood at an average age of around 13 years.^1,2^ A recent population-based study in Ontario, Canada, found an age-standardized incidence and prevalence of pediatric multiple sclerosis in 2014 of approximately 1.24/100 000 and 6.8/100 000 children, respectively.^3^

Although pediatric multiple sclerosis is therefore uncommon, children face a high burden of disease, including a high rate of clinical relapse, cognitive impairment, and the occurrence of disability and progressive disease at a younger age than those with adult-onset multiple sclerosis.^1,2,4,5^ First-line disease-modifying therapies for children with multiple sclerosis have typically been injectable agents, namely, interferons or glatiramer acetate.^6,7^ However, efficacy with injectable agents is less than other agents currently available, and many children who begin therapy with an injectable agent will eventually switch to another disease-modifying therapy.^8,10^ Only recently was the first phase 3 randomized controlled trial of a disease-modifying therapy in children with multiple sclerosis completed, resulting in fingolimod becoming the first disease-modifying therapy approved by Health Canada for use in children with multiple sclerosis in 2018.^11^ The treatment of pediatric multiple sclerosis is thus evolving, with a shift toward earlier use of higher efficacy therapies.^12^ However, there has been no Canadian data published on how the approval of fingolimod and shifting treatment philosophy has altered disease-modifying therapy prescribing patterns in children with multiple sclerosis.

The province of Alberta has one of the highest overall rates of multiple sclerosis in Canada and so the incidence and prevalence of multiple sclerosis in Albertan children are expected to be at least as high, if not higher, than that found in Ontarian children.^13,14^ In this study, we aim to describe trends in the incidence and prevalence of pediatric multiple sclerosis in Alberta over the past 10 years, along with evolutions in patterns of disease-modifying therapy prescribing.

## Materials and Methods

### Data Sources

This study entailed a retrospective review of administrative health databases maintained by the province of Alberta, Canada, which administers a universal publicly funded health system to a population of approximately 4.4 million residents. The population registry contains basic demographic information along with dates of active health insurance coverage, including information on death and in- and out-migration. The discharge abstract database contains information related to inpatient hospital admissions including dates of service and discharge diagnoses by *International Classification of Diseases, Tenth Revision, Canada* (*ICD-10-CA*) codes. The practitioner claims database contains information related to physician services (both fee-for-service and shadow-billing) including date of service and physician-assigned diagnosis by *International Classification of Diseases, Ninth Revision* (*ICD-9*) code. The pharmaceutical information network contains information pertaining to pharmaceutical dispenses including the date of dispense and drug information (by Drug Identification Number [DIN] and Anatomic Therapeutic Classification [ATC] code), with approximately 95% of pharmacies in the province contributing records. A unique patient identifier was used to link subjects across data sets. Data encompassing the period from January 1, 2006, to December 31, 2020, were included.

### Case Definitions

We compared 2 administrative case definitions of multiple sclerosis that have previously been validated in a pediatric cohort in Ontario, a Canadian province with a publicly funded health system similar to that of Alberta.^3^ The Marrie definition requires ≥3 hospitalizations or physician claims associated with an multiple sclerosis diagnosis (*ICD-9* code 340, *ICD-10-CA* code G35) in all available years of data.^3^ The Canadian Chronic Disease Surveillance System (CCDSS) definition requires ≥1 hospitalization or ≥5 physician claims associated with a multiple sclerosis diagnosis within 2 years.^15^ To avoid double-counting, multiple physician claims within the same day were counted as a single claim, and physician claims overlapping a hospitalization associated with a multiple sclerosis diagnosis were excluded. Hospitalizations beginning within  ±1 day of another hospital discharge were considered part of the same hospitalization to avoid double-counting related to transfer between health care facilities. Those patients <19 years of age at first documentation of a multiple sclerosis diagnosis were included.

### Incidence and Prevalence

In those patients fulfilling the case definition for multiple sclerosis, the date of diagnosis (index date) was defined as the earliest hospital visit/physician claim associated with a central nervous system demyelinating diagnosis (*ICD-9* / *ICD-10-CA*: multiple sclerosis [340/G35], optic neuritis [377.3/H46], acute transverse myelitis [323.6, 323.8, 323.9, 341.2/G37.3], acute disseminated encephalomyelitis [323.6, 323.8, 323.9/G04.0], neuromyelitis optica [341.0/G36.0], other demyelinating diseases of the central nervous system [341/G37], or other acute disseminated demyelination [G36]). Cases were considered incident at the time of the index date if there were no claims for a demyelinating disease diagnosis in the 5 years before the index date. Cases were deemed prevalent from the index date through all subsequent years in which active provincial health insurance coverage was maintained.

The period for incidence and prevalence calculations was the fiscal year (April 1–March 31), with the population having active provincial health insurance coverage at the fiscal year-end used as the denominator. Prevalent multiple sclerosis cases from prior years were excluded from the population denominator when calculating incidence. Crude incidence and prevalence estimates were calculated from 2011-2012 (fiscal year-end 2012) through 2019-2020 (fiscal year-end 2020) and 95% confidence intervals were calculated using the exact method. Incidence and prevalence were also stratified by sex and age cohort (0-11 years, 12-15 years, 16-18 years). Age-standardized incidence and prevalence were calculated using data from the 2016 Canadian census as the standard population, and 95% confidence intervals were calculated based on a gamma distribution.

### Disease-Modifying Therapies

Pharmaceutical data were analyzed for those patients fulfilling either case definition for multiple sclerosis. Pharmacy dispenses of disease-modifying therapies during the period January 1, 2011–December 31, 2020, were identified by ATC code and classified as injectables (glatiramer acetate, interferon-beta) or newer agents (fingolimod, teriflunomide, dimethyl fumarate, cladribine, natalizumab, ocrelizumab, rituximab, alemtuzumab). Patterns of disease-modifying therapy use were analyzed including the agent(s) dispensed, age at disease-modifying therapy dispense, time to first disease-modifying therapy dispense from the index date (days), and the number of unique disease-modifying therapies dispensed per patient during the study period. Where more than 1 unique disease-modifying therapy was dispensed for an individual, the days between the initial dispenses of each agent were calculated.

### Data Analysis

Continuous variables were summarized as median (interquartile range [IQR]). The time from the index date to the first documented disease-modifying therapy dispense and the time between the first and second disease-modifying therapy dispenses (when applicable) were compared between patients with an index date in the early (2011-2015) versus later (2016-2020) halves of the study period using the Mann-Whitney *U* test. *P* values <.05 were considered significant. Data analysis was performed using R version 4.1.0. and the epiR package.^16,17^

## Results

### Eligibility

Between 2011 and 2020, a total of 106 children were identified as meeting one or both administrative case definitions of multiple sclerosis. Ninety-nine children (69 female) met the Marrie definition, 80 children (58 female) met the Canadian Chronic Disease Surveillance System definition, and 73 children (53 female) fulfilled the criteria for both definitions.

### Incidence

**Figure 1.**
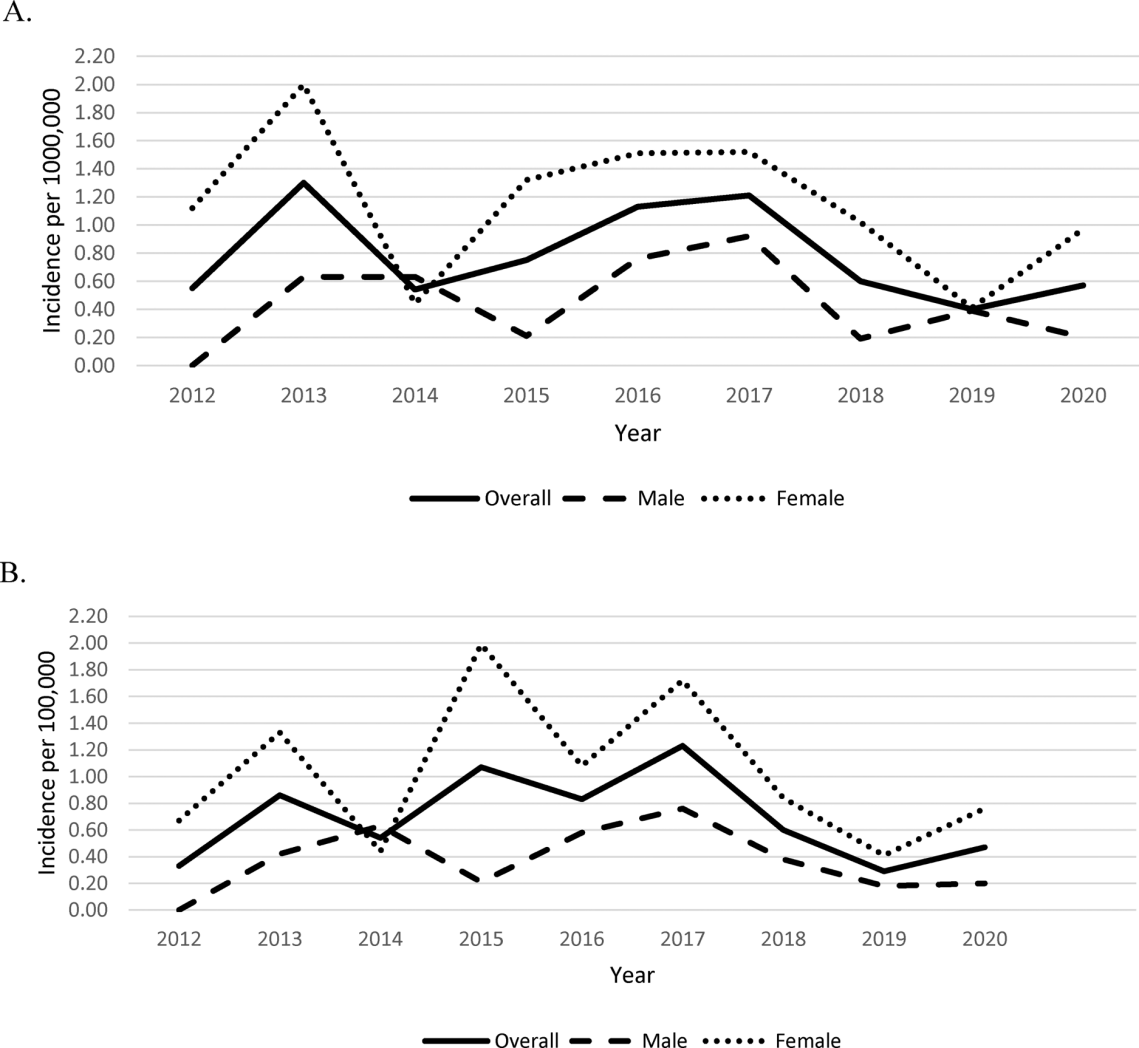
Annual age-standardized incidence of multiple sclerosis <19 years of age per 100 000 population, as determined by the (A) Marrie definition and (B) Canadian Chronic Disease Surveillance System (CCDSS) definition.

From 2011 to 2020, a total of 79 incident cases were identified meeting either case definition, with a median age at index date of 16 years (IQR 13.5-17). According to the Marrie definition, 72 incident cases of pediatric-onset multiple sclerosis were identified, resulting in an age-standardized annual incidence of 0.55 (95% CI 0.18-1.28) per 100 000 in 2012 compared with 0.57 (95% CI 0.21-1.26) per 100 000 in 2020 (Figure 1A).

Using the Canadian Chronic Disease Surveillance System definition, a total of 64 incident cases were identified during the study period. The age-standardized annual incidence determined by the Canadian Chronic Disease Surveillance System definition was 0.33 (95% CI 0.07-0.96) per 100 000 in 2012 compared to 0.47 (95% CI 0.15-1.12) per 100 000 in 2020 (Figure 1B).

Age-standardized incidence according to both definitions was higher in females in all study years except for 2014. In 2020, the incidence of pediatric-onset multiple sclerosis in females using the Marrie definition was 0.97 (95% CI 0.31-2.29) per 100 000 compared to 0.20 (95% CI 0.01-1.11) per 100 000 in males. Using the CCDS definition, the incidence in 2020 was 0.76 (95% CI 0.21-1.99) per 100 000 in females compared to 0.20 (95% CI 0.01-1.11) per 100 000 in males.

### Prevalence

**Figure 2.**
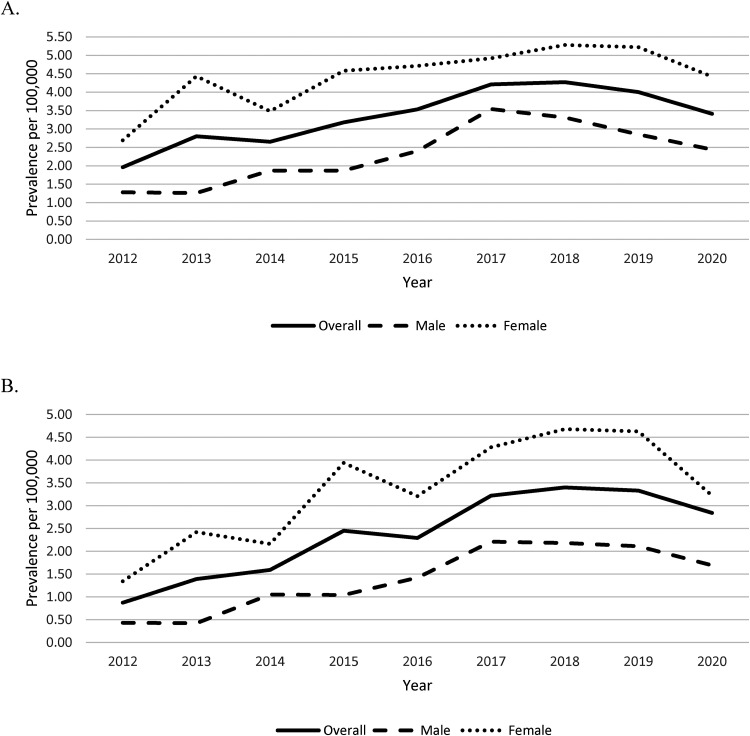
Annual age-standardized prevalence of multiple sclerosis <19 years of age per 100 000 population as determined by the (A) Marrie definition and (B) Canadian Chronic Disease Surveillance System (CCDSS) definition.

Using the Marrie definition, the age-standardized annual prevalence of multiple sclerosis increased from 1.96 (95% CI 1.16-3.11) per 100 000 in 2012 to 3.41 (95% CI 2.37-4.75) per 100 000 in 2020 (Figure 2A). In 2020, this corresponds to 35 children living with multiple sclerosis in Alberta.

According to the CCDS definition, the age-standardized annual prevalence of multiple sclerosis increased from 0.87 (95% CI 0.38-1.72) per 100 000 in 2012 to 2.84 (95% CI 1.90-4.09) per 100 000 in 2020 (Figure 2B).

Age-standardized prevalence of pediatric-onset multiple sclerosis was greater in females in all study years. In 2020, the prevalence using the Marrie definition was 4.42 (95% CI 2.77-6.70) per 100 000 in females compared to 2.44 (95% CI 1.30-4.20) per 100 000 in males. Using the CCDS definition, the prevalence in 2020 was 3.22 (95% CI 1.84-5.25) per 100 000 in females compared to 1.69 (95% CI 0.77-3.24) per 100 000 in males.

### Disease-Modifying Therapies

Of the 106 children fulfilling at least 1 case definition for multiple sclerosis, 61 (58%) filled a prescription for at least 1 disease-modifying therapy during the study period, including 61 of 99 (62%) fulfilling the Marrie case definition and 57 of 80 (71%) fulfilling the CCDS case definition. Forty-nine children had a first disease-modifying therapy dispense <19 years of age. The youngest age at first disease-modifying therapy dispense was 7 years, and only injectables were dispensed to those <13 years of age. The median number of unique disease-modifying therapies dispensed over the study period for all participants with ≥1 disease-modifying therapy dispense was 1.6 (range 1-4). Of 33 prevalent cases <19 years of age at FYE 2015, 7 (21%) were dispensed a disease-modifying therapy that year compared to 16 of 37 (43%) prevalent cases dispensed a disease-modifying therapy in FYE 2020.

Of the 79 multiple sclerosis cases deemed incident during the study period, 47 (59%) had at least 1 disease-modifying therapy dispense (Table 1), with a median age at the first dispense of 17.2 years (IQR 16.0-18.6). The median time from the index date to the first disease-modifying therapy dispense was 263 days (IQR 134.5-988). In those who were dispensed more than 1 unique disease-modifying therapy, the median time from the first dispense of the initial disease-modifying therapy to the first dispense of the second disease-modifying therapy was 567 days (IQR 267-927). When the first and second portions of the study period were compared, the median time from the index date to the first disease-modifying therapy dispense decreased from 424 days (IQR 142-1409.5) in 2011-2015 to 186 days (IQR 133-417) in 2016-2020 (*P*  =  .06).

**Table 1.** Clinical Information for Incident Cases, by Year of Incidence.

	2011-2020	2011CSBOLDSTART-2015CSBOLDEND	2016-2020	*P* value
INCIDENT CASES, n	79	41	38	–
FEMALE, N (%)	57 (72)	31 (76)	26 (68)	–
AGE COHORT AT INCIDENCE, N				–
0-11 Y	9	4	5	
12-15 Y	28	14	14	
16-18 Y	42	23	19	
NUMBER OF DMTs, N				–
0	32	15	17	
1	26	12	14	
2	16	9	7	
≥3	5	5	0	
FIRST DMTCSBOLDSTART DISPENSED <19 Y OF AGE, NCSBOLDEND				–
INJECTABLE	26	15	11	
Newer	12	3	9	
TIME FROM INDEX DATE TO INITIAL DMTCSBOLDSTART DISPENSECSBOLDEND, d, median (IQR)	263 (134.5-988)	424 (142-1409.5)	186 (133-417)	.06
TIME FROM FIRST TO SECOND DMTCSBOLDSTART DISPENSE, DCSBOLDEND, MEDIAN (IQR)	567 (267-927)	624.5 (318.5-1268.5)	400 (266.5-551)	.21

Abbreviations: DMT, disease-modifying therapy; IQR, interquartile range.

**Figure 3.**
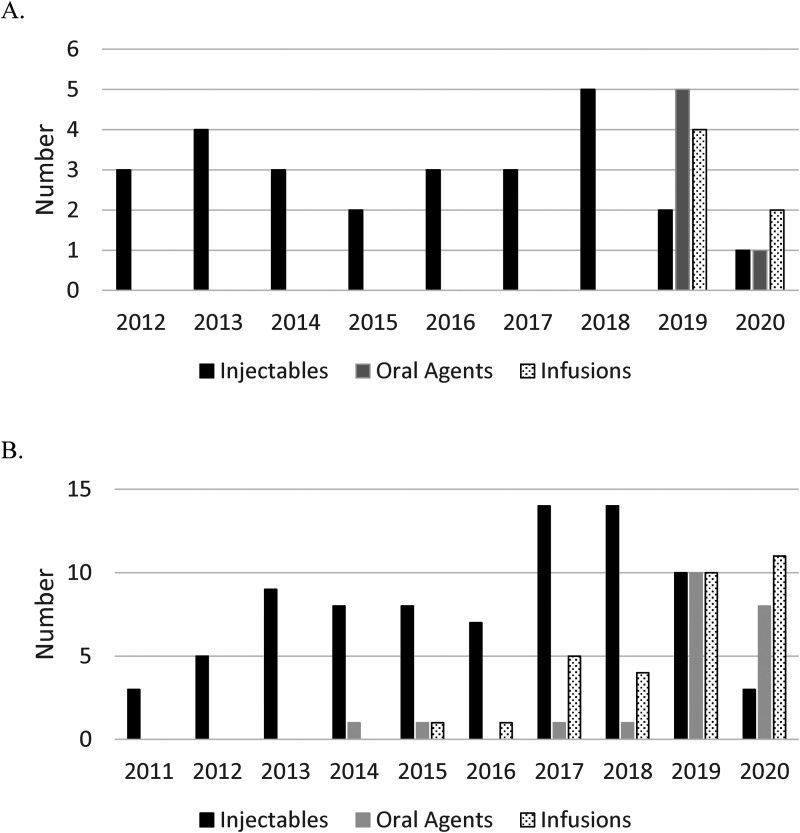
(A) First disease-modifying therapy dispensed <19 years of age in those cases classified as incident during the study period, categorized by year of the first dispense and medication class (injectables, oral agents, and infusions). (B) Unique disease-modifying therapy dispenses <19 years of age in all prevalent cases, categorized by year of dispense and medication class (injectables, oral agents, and infusions).

Thirty-eight incident cases were initially dispensed a disease-modifying therapy prior to 19 years of age, with injectables accounting for the majority of initial disease-modifying therapy dispenses in children over the study period (26/38, 68%). Injectables accounted for all first disease-modifying therapy dispenses before 2019, whereas from 2019-2020 injectables accounted for only 3 of 15 (20%) initial dispenses (Figure 3A). Glatiramer acetate accounted for the majority of initial injectable dispenses (n  =  18), compared to interferons (n  =  8). The most common newer disease-modifying therapy dispensed as initial therapy in children was rituximab (n  =  5), followed by dimethyl fumarate (n  =  3), fingolimod (n  =  2), ocrelizumab (n  =  1), and cladribine (n  =  1).

Most children (17/26, 65%) initially dispensed an injectable disease-modifying therapy were later dispensed an alternate disease-modifying therapy. Conversely, only 1 of 12 (8%) children initially dispensed a newer disease-modifying therapy switched during the study period. No children initially dispensed an infusion therapy were dispensed an alternate disease-modifying therapy during the study period. Including unique disease-modifying therapies dispensed for all prevalent cases <19 years of age, the most common disease-modifying therapies dispensed in 2020 were fingolimod (n  =  6) and rituximab (n  =  5), followed by ocrelizumab (n  =  4), glatiramer acetate (n  =  3), dimethyl fumarate (n  =  2), and natalizumab (n  =  2) (Figure 3B).

## Discussion

In this retrospective study, we used administrative health data to define the incidence and prevalence of pediatric-onset multiple sclerosis in Alberta and describe changes in disease-modifying therapy prescribing over time. Using the Marrie case definition, the age-standardized incidence and prevalence of pediatric-onset multiple sclerosis was 0.57/100 000 and 3.41/100 000, respectively, in 2020. With the use of the Canadian Chronic Disease Surveillance System definition for multiple sclerosis, the age-standardized incidence and prevalence of pediatric-onset multiple sclerosis was 0.47 per 100 000 and 2.84 per 100 000, respectively, in 2020. These results are consistent with the Marrie definition being more sensitive than the Canadian Chronic Disease Surveillance System definition for detecting multiple sclerosis cases.^3^

Compared with Ontario data, in 2014 (the last comparable year between the 2 studies) the incidence and prevalence of pediatric multiple sclerosis was 1.22/100 000 and 6.8/100 000 in Ontario versus 0.54/100 000 and 2.65/100 000 in our study.^3^ International studies have reported an incidence of pediatric multiple sclerosis ranging from 0.26/100 000 in the Netherlands to 2.85/100 000 in Sardinia.^18,22^ Prevalence estimates have ranged from to 0.69/100 000 in Japan to 26.92/100 000 in Sardinia.^19,20,23^ The incidence and prevalence of pediatric multiple sclerosis in Alberta is thus within the range of that reported in other jurisdictions but is not among the highest, contrary to what has been previously reported in the general population of Alberta.^13,14^ However, direct comparison of incidence and prevalence data with other jurisdictions is limited by variable methods in identifying cases between studies, including surveys, hospital data, and national multiple sclerosis registries. Although we did not include pharmacy data in our case definition to allow a more direct comparison with Ontario data, it is possible that inclusion of this information in the case definition may have further increased its sensitivity.

Notably, only 58% of subjects meeting an multiple sclerosis case definition in our study were dispensed a disease-modifying therapy during the study period. It is possible that some disease-modifying therapy use was missed—for example, disease-modifying therapies dispensed in the inpatient setting, in the context of clinical trials, or in pharmacies not contributing administrative data—although this would not be expected to be a large number. Conversely, this could reflect false positives in the multiple sclerosis case definition and suggest a lower positive predictive value of the criteria in Alberta compared to the >90% positive predictive value reported in Ontario.^3^ Finally, this could indicate that many cases of pediatric-onset multiple sclerosis in Alberta are not being appropriately treated with a disease-modifying therapy. For comparison, a prospective study of pediatric multiple sclerosis centers in the United States reported that 78.5% of children with multiple sclerosis received a disease-modifying therapy before age 18 years.^7^

Through pharmacy data, we identified a significant evolution in the treatment of pediatric-onset multiple sclerosis in Alberta over time. Beginning in 2019, there has been a dramatic shift away from the use of injectables as first-line or ongoing therapy, such that only a small number of children were dispensed an injectable disease-modifying therapy in 2020. A similar shift toward newer agents has been reported by US pediatric multiple sclerosis centres,^7^ although the shift in Alberta has occurred chronologically later and more rapidly. Multiple reasons may be behind this shift, including evidence from the PARADIGMS trial supporting the efficacy of fingolimod over interferon beta-1a,^11^ evidence supporting the benefit of higher efficacy disease-modifying therapies in pediatric multiple sclerosis,^24^ increasing data in adult multiple sclerosis populations regarding the safety and efficacy of newer agents,^25,27^ and changes in health insurance coverage. Importantly, this study provides the first Canadian data on how the treatment of children with multiple sclerosis may have changed following Health Canada's first approval of a disease-modifying therapy in the pediatric population (fingolimod). Although fingolimod dispenses did increase after its approval in 2018, it continues to account for less than one-third of disease-modifying therapy dispenses despite being the only current Health Canada approved disease-modifying therapy for children with multiple sclerosis. B-cell therapies—rituximab and ocrelizumab—are now the most common class of disease-modifying therapy dispensed in Alberta for children with multiple sclerosis, both as initial therapy and overall. Thus, there continues to be a significant treatment need that is not being met by fingolimod, although this study cannot comment on the role that clinician factors, patient/family factors, or others (such as drug coverage) may be playing in these treatment patterns. This highlights the ongoing need for clinical trials and other studies of newer multiple sclerosis therapies in children to better establish their safety and efficacy in the pediatric population and to ensure reliable access to these therapies, particularly as they are being prescribed more commonly. No patients dispensed a B-cell therapy were subsequently dispensed an alternate disease-modifying therapy, consistent with the low discontinuation rate reported in previous studies.^26,27^ However, given that most B-cell therapies were dispensed toward the end of our study period, longer-term follow-up is required to adequately assess the persistence on B-cell therapies for pediatric-onset multiple sclerosis in Alberta.

Our results also identified a trend toward earlier initiation of disease-modifying therapies in children with multiple sclerosis, decreasing from a median time of 424 days from index date in the 2011-2015 period to 186 days in those diagnosed from 2016-2020. This is encouraging given the increased recognition of the importance of early disease-modifying therapy use—including the early use of high-efficacy therapies—in reducing the risk of long-term disability in multiple sclerosis.^28,29^ Future studies will be required to assess whether this shift toward earlier use of higher-efficacy agents for pediatric multiple sclerosis in Alberta will result in long-term improvements in clinical or health economic outcomes.

The strengths of this study include the use of administrative data sets from a universal, publicly funded health care system that are expected to reliably capture the epidemiology and pharmaceutical utilization of children living with multiple sclerosis in Alberta. Furthermore, the case definitions used to identify children with multiple sclerosis have been previously validated elsewhere in Canada and are expected to perform similarly in Alberta given similarities in health care systems.^3,15^ The use of administrative health data is also a limitation, as individual medical records were not reviewed to confirm the diagnosis of multiple sclerosis nor to analyze additional information related to clinical outcomes, drug safety, or efficacy.

Overall, we found the incidence and prevalence of pediatric-onset multiple sclerosis in Alberta to be within the range previously described in other jurisdictions, although not among the highest reported. Based on these results, there are an estimated 35 children living with multiple sclerosis in Alberta as of 2020. There has been a rapid shift in recent years away from injectable disease-modifying therapies, with B-cell therapies now being the most common disease-modifying therapy category dispensed for children with multiple sclerosis in Alberta. Longer-term follow-up of this population is required to determine if this shift in treatment approach is resulting in improved clinical, health economic, or socioeconomic outcomes or, conversely, adverse safety events.
